# Dataset regarding baseline and follow-up characteristics of out-of-hospital cardiac arrest patients focused on neurological outcomes

**DOI:** 10.1016/j.dib.2018.10.086

**Published:** 2018-10-27

**Authors:** Juan Caro-Codón, Juan R. Rey, Esteban Lopez-de-Sa, Óscar González Fernández, Sandra O. Rosillo, Eduardo Armada, Ángel M. Iniesta, Jaime Fernández de Bobadilla, José Ruiz Cantador, Laura Rodríguez Sotelo, Francisco Javier Irazusta, Verónica Rial Bastón, Pablo Merás Colunga, José Luis López-Sendón

**Affiliations:** Cardiology Department, Hospital Universitario La Paz, Madrid, Spain

## Abstract

This data article contains the data related to the research article entitled “Long-term neurological outcomes in out-of-hospital cardiac arrest patients treated with targeted-temperature management” (Caro-Codón et al., 2018). In this dataset, we report details regarding the flow chart of the included patients and the specific exclusion criteria. We also include information on the difference between the patients who attended the structured personal interview (and therefore were finally included in the study) and those who did not attend. Neuropsychiatric and functional data before and after cardiac arrest are also reported. Finally, we list all the “de novo” focal neurological deficits identified after cardiac arrest in the related population.

**Specifications table**

**Table:** 

Subject area	*Acute cardiac care.*
More specific subject area	*Out-of-hospital cardiac arrest outcomes.*
Type of data	*Figure and tables*
How data were acquired	*Structured personal interview, neurocognitive tests, review of clinical records.*
Data format	*Analyzed.*
Experimental factors	*Personal interview and realization of the following cognitive tests: MoCA, Trail making test, EuroQoL-5D-3L, Modified IQCODE, Zarit Caregiver Burden Interview, CPC, modified Rankin scale.*
Experimental features	*Creation of an specific database and statistical analysis using dedicated software (Stata v. 14, StataCorp).*
Data source location	*Tertiary care hospital in Madrid, Spain.*
Data accessibility	*Data are with this article*
Related research article	*Data on long-term neurological outcomes in out-of-hospital cardiac arrest patients treated with targeted-temperature management. J. Caro-Codón et al. Resuscitation 133 (2018) 33–39.*

**Value of the data**

• The data included in this dataset can be used by the medical community to make an adequate assessment of the long-term prognosis in survivors after out-of-hospital cardiac arrest. Major problems related with OHCA in contemporary acute cardiac care are in the below points:
• Mortality after cardiac arrest remains very high, even if the patient achieve return of spontaneous circulation.
• Hypoxic-ischemic brain injury is the leading cause of death in OHCA patients.
• Information regarding long-term neurological prognosis is lacking.
• Most commonly used clinical scales to assess neurological outcomes in these patients are crude and lack sensitivity to detect mild or moderate cognitive deficits.

## 1 Data

**Fig. 1:**
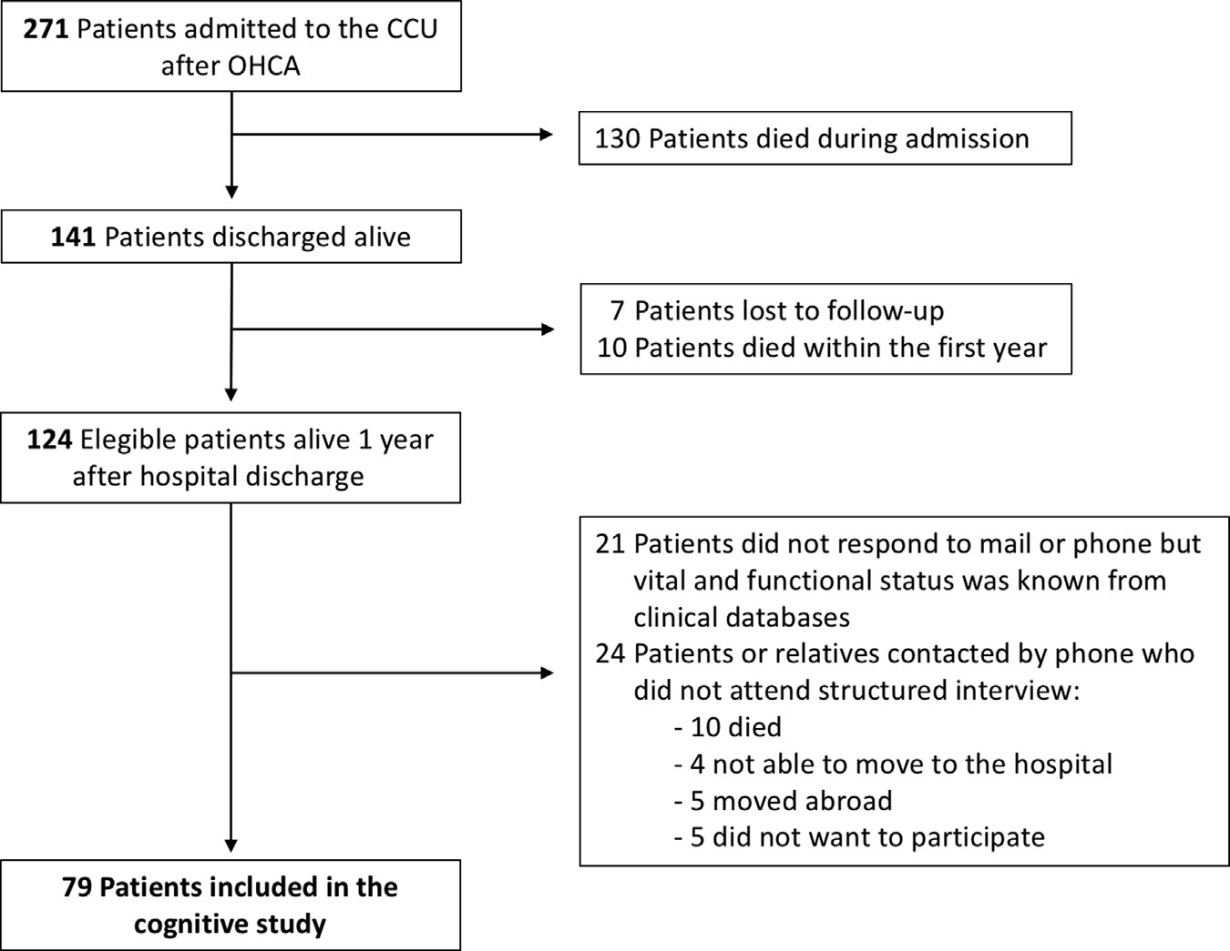
Flow chart for patients admitted to the CCU who were comatose survivors of an out-of-hospital cardiac arrest (OHCA) during the study period.

The data provided in this data article compliment the original research article describing the long-term outcomes of a selected population of out-of-hospital cardiac arrest patients surviving at least one year after the index event. Fig. 1 illustrates the flow chart of the study population, describing how many patients were finally excluded from the analysis and the detailed exclusion causes. Table 1 describes the characteristics of the patients who attended the personal interview compared to those who did not attend. Table 2 summarize the difference between neuropsychiatric and functional characteristics of the patients at baseline and during follow-up. Table 3 describe the ‘de novo’ focal neurological deficits identified after cardiac arrest and list the corresponding radiological findings.

**Table 1:** Characteristics of the patients who attended the personal interview and were included in the study compared to those who did not attend.

	Not included (*n *= 45)	Included (*n *= 79)	*p*-value
Male sex	37 (82.2%)	71 (89.9%)	0.22
Age, mean±SD, years	62.2 ± 14.6	53.5 ± 14.5	0.002
History of ischemic heart disease	13 (28.9%)	13 (16.5%)	0.11
Atherosclerosis^*^	18 (40%)	19 (24.1%)	0.062
Previous stroke	5 (11.1%)	3 (3.8%)	0.14
Witnessed cardiac arrest	42 (93.9%)	76 (96.2%)	0.67
Initial shockable rhythm	35 (77.8%)	71 (89.9%)	0.066
Time to ROSC, median (IQR), minutes	18.0 (12.0–24.5)	17.0 (12.0–26.0)	0.37
Time from CA to initiation of CPR, median (IQR), minutes	2.0 (0.0–6.0)	2.0 (1.0–5.0)	0.18
First documented pH, median (IQR)	7.22 (7.11–7.27)	7.23 (7.09–7.32)	0.44
First documented bispectral index, median (IQR)	34 (0–47)	40 (15–50.5)	0.19
Targeted temperature			
32 °C	16 (35.6%)	31 (39.2%)	0.78
33 °C	24 (53.3%)	42 (53.2%)	
34 °C	5 (11.1%)	6 (7.6%)	
Time to awakening, median (IQR), hours	67.8 (54.3–102.8)	60.7 (46.9–92.4)	0.19
EF at hospital discharge	41.2 ± 14.9	48.9 ± 14.1	0.005
ICD implantation	15 (33.3%)	37 (46.8%)	0.14

**Table 2:** Neuropsychiatric and functional characteristics before and after cardiac arrest.

	Before CA	After CA	*p*-value
Married	62 (78.5%)	61 (77.2%)	*p* = 0.84
Live with someone else	70 (88.6%)	70 (88.6%)	*p* = 1.0
Memory disorders*	0 (0%)	34 (43%)	*p* < 0.001
Depression symptoms	14 (17.7%)	28 (35.4%)	*p* = 0.001
Use of antidepressant drugs	4 (5.1%)	16 (20.3%)	*p* < 0.001
Emotional lability	0 (0%)	7 (8.9%)	*p *= 0.013
Neurological focal deficits	1 (1.3%)	10 (12.7%)	*p* = 0.005
Behavioral disorders**	0 (0%)	10 (12.7%)	*p* = 0.002
Employment situation			
Full-time work	54 (68.4%)	34 (43.0%)	*p* < 0.001
Part-time work	0	3 (3.8%)	
Inferior work position	N/A	4 (5.1%)	
Unemployed	5 (6.3%)	6 (7.6%)	
Medically unfit	2 (2.5%)	14 (17.7%)	
Retired	18 (22.8%)	18 (22.8%)	
Driver	60 (75.9%)	54 (68.4%)	*p *= 0.013
Sport activity, median (IQR), hours/week	0 (0–3)	0 (0–4)	*p* = 0.99
Light physical activity, median (IQR), hours/week***	5 (2–7)	6 (4–9)	*p* = 0.034
Intellectual activity, median (IQR), hours/week+	7.3 (5–14)	7.3 (5–14)	*p* = 0.87

Abbreviations: CA cardiac arrest.

* Referred by the patient himself or a reliable informant.

** Behavioral disorders include aggressive and/or uninhibited behaviors.

*** Light physical activity refers to activity with metabolic requirements of less than 4 METS (e.g., walking, shopping, domestic activities …).

+ Includes activities such as reading, using the internet, watching movies, listening to news, educational activities.

**Table 3:** De novo focal neurological deficits after cardiac arrest.

Patient	Clinical symptoms	Radiological findings
1	Dysarthria and spasticity	Corona radiata and basal ganglia lesions (CT)
2	Anosmia and ageusia	No pathological findings (CT)
3	Ataxia	Cortical retraction. Dilatation of the Virchow-Robin spaces (MR)
4	Diplopia	Lacunar infarction involving the III cranial nerve nucleus (MR)
5	Dystonias	Cortical retraction. Dilatation of the Virchow-Robin spaces (MR)
6	Bilateral neurosensorial hearing loss	No neurological imaging tests performed
7	Unilateral neurosensorial hearing loss	No pathological findings (CT)
8	Ataxia	No pathological findings (CT)
9	Ischemic optic neuropathy	No neurological imaging tests performed

## 2 Experimental design, materials, and methods

### 2.1 Patients

Comatose patients admitted to the Acute Cardiac Care Unit after OHCA, from August 2007 to November 2015 and surviving at least one year after the index event were included. All patients received targeted-temperature management according to the current protocol in our center. The targeted temperature (32–34 °C) was either selected at discretion of the treating physician or assigned by randomization as part of a clinical trial [2], [3]. Patients with open cognitive impairment (CPC 3–4) prior to the event were excluded.

### 2.2 Study protocol

The study protocol was approved by the Institutional Ethics Committee. We prospectively contacted all patients who met inclusion criteria, arranging a face-to-face interview. They were invited to come accompanied by a reliable informant. Each patient completed a structured interview focused on the collection of clinical, social and demographic data. All available information in clinical records was reviewed and a battery of neurocognitive and psychometric tests was performed.

### 2.3 Specific evaluation

Details on the specific cognitive tests used during the structured interview (MoCA, TMT part B, EuroQoL-5D-3L, modified IQCODE, Zarit Caregiver Burden Interview, cerebral performance category and modified Rankin scale) can be found in the related research article [1].

### 2.4 Statistical analysis

Categorical variables are presented as counts and percentages, and were compared using the χ^2^ test or Fisher exact test. Continuous variables are presented as mean±SD or medians and interquartile ranges. They were analyzed using t tests or the Mann-Whitney U test. Forward stepwise logistic regression analysis was used to build a predictive model selecting as the dependent variable the existence of cognitive impairment according to the MoCA test. We included in the set of possible explanatory variables those that were statistically significant in the univariate analysis and other prognostic factors previously identified in other related investigations. All data were analyzed using the statistical package Stata v14.2 (StataCorp, College Station, TX, USA).
