# Supplementary material for: Dataset regarding baseline and follow-up characteristics of out-of-hospital cardiac arrest patients focused on neurological outcomes
Source: Data Brief. 2018 Oct 27;21:1140–4. doi: 10.1016/j.dib.2018.10.086 (PMC6231031; doi:10.1016/j.dib.2018.10.086)
Supplement: Supplementary file 1 — Supplementary material. [file mmc1.docx]

**CONFLICT OF INTEREST STATEMENT**

All the authors wish to confirm that there are no known conflicts of interest associated with this publication and there has been no significant financial support for this work that could have influenced its outcome.
